# Risk and Protective Factors of Lifetime Cocaine-Associated Chest Pain

**DOI:** 10.3389/fpsyt.2021.704276

**Published:** 2021-07-21

**Authors:** Virgile Clergue-Duval, Louise Nicolas-Sacy, Emily Karsinti, El-Hadi Zerdazi, Jean-Louis Laplanche, Georges Brousse, Andries T. Marees, Eske M. Derks, Patrick Henry, Frank Bellivier, Florence Vorspan, Vanessa Bloch

**Affiliations:** ^1^APHP, Département de Psychiatrie et de Médecine Addictologique, Site Lariboisière Fernand-Widal, Groupe hospitalier universitaire APHP Nord - Université de Paris, Paris, France; ^2^Inserm UMRS-1144 Optimisation Thérapeutique en Neuropsychopharmacologie, Université de Paris, Paris, France; ^3^Fédération Hospitalo-Universitaire NOR-SUD Network of Research in Substance Use Disorders, Ile-de-France, France; ^4^Faculté de Médecine, Université de Paris, Paris, France; ^5^APHP, Pharmacie, Site Lariboisière Fernand-Widal, Groupe Hospitalier Universitaire APHP Nord - Université de Paris, Paris, France; ^6^Laboratoire ClipsyD, Université Paris Nanterre, Nanterre, France; ^7^APHP, Service d'addictologie, DMU IMPACT, GHU Mondor, Hôpital Emile ROUX, Limeil Brévannes, France; ^8^APHP, Département de Biochimie et Biologie Moléculaire, Site Lariboisière Fernand-Widal, Groupe Hospitalier Universitaire APHP Nord - Université de Paris, Paris, France; ^9^Faculté de Pharmacie, Université de Paris, Paris, France; ^10^Service de psychiatrie-addictologie, CHU de Clermont-Ferrand, Université Clermont-Auvergne, Clermont-Ferrand, France; ^11^Department of Economics, School of Business and Economics, VU University Amsterdam, Amsterdam, Netherlands; ^12^Queensland Institute of Medical Research Berghofer, Translational Neurogenomics Group, Brisbane, QLD, Australia; ^13^APHP, Département de Cardiologie, Site Lariboisière Fernand-Widal, Groupe Hospitalier Universitaire APHP Nord - Université de Paris, Paris, France

**Keywords:** cocaine, chest pain, acute coronary syndrome, opioid maintenance treatment, single nucleotide polymorphism, *GUCY1A3*, *ALDH2*, *rs2238151*

## Abstract

**Introduction:** Cocaine users often present with repetitive events of cocaine-associated chest pain (CACP), clinically resembling acute coronary syndromes. The aim of the study is to describe the specific risk factors for CACP.

**Method:** Cocaine users (*n* = 316) were recruited for a multicenter cross-sectional study. Lifetime CACP history, sociodemographic factors, and lifetime use of cocaine and other substances were assessed. Thirty single nucleotide polymorphisms (SNPs) of NOS3, ROCK2, EDN1, *GUCY1A3*, and *ALDH2* genes, suggested by the literature on coronary spasms, were selected. The associations with CACP history were tested using the chi-square test, Student's *t*-test and logistic regression.

**Results:** Among the 316 subjects [78.5% men, mean age 37.5 years, (standard-deviation ±8.7)], 190 (60.1%) were daily cocaine users and 103 (32.6%) reported a lifetime CACP history. Among those with a lifetime CACP history, the median was 10 events per individual. In multivariate analysis, lifetime CACP history was associated with daily cocaine use [odds-ratio (OR) 3.24; 95% confidence intervals (1.29–9.33)], rapid route of cocaine use [OR 2.33 (1.20–4.64) vs. intranasal use], and lifetime amphetamine use [daily amphetamine use: OR 2.80 (1.25–6.32) and non-daily amphetamine use: OR 2.14 (1.15–4.04) vs. never used]. Patients with lifetime opioid maintenance treatment (OMT) reported significantly less lifetime CACP history [OR 0.35 (0.16–0.76)]. None of the selected SNPs was associated with CACP history after multiple testing corrections.

**Conclusions:** Clinical variables describing the intensity of stimulant use were positively associated with lifetime CACP history, while OMT was negatively associated with it. Specific harm reduction strategies can target these risk factors.

## Introduction

Cocaine use is prevalent, and according to the estimates, 18 million individuals declared use of cocaine during the past year worldwide (1). Cocaine is also the fifth most consumed psychoactive substance in Europe (after alcohol, tobacco, benzodiazepine, and cannabis) and the sixth in the United States (also after opioids) (2, 3). In Europe, the average rate of cocaine use in the past year was 1.2% among individuals aged 15–64 years and 2.1% among those aged 15–34 years (3). In France, in 2017, 2.8% of 18–25-year-old and 3.4% of 26–34-year-old individuals had used cocaine in the previous year (4).

Cocaine has a direct cardiac effect and induces several cardiovascular complications, including myocardial ischemia and infarction, acute coronary syndrome, aortic dissection, heart failure, hypertension, arrhythmias, and stroke (5–8). According to a recent study, 17.6% of patients who visited emergency departments after cocaine consumption were affected by chest pain (9). This was the third most frequent complaint after anxiety (32.2%) and agitation or aggressive behavior (27.4%) (9). Cocaine-associated chest pain (CACP) accounts for 1.9–6.9% of all chest pain consultations in emergency departments (10, 11). Among young people, these percentages are even higher, with a 25% prevalence of cocaine use among patients younger than 30 years old (10). Of note, a quarter of patients with CACP will return to the emergency department for a similar chest pain symptom during the following year (12). Indeed, lifetime CACP is reported by half of the cocaine users who received treatment in an addiction treatment center (11), and repetitive episodes are frequent (11–14). CACP is characterized by symptoms similar to those of acute coronary syndrome, including constrictive or oppressive retrosternal pain (11, 13, 15). However, <6% of patients with transient CACP have myocardial infarction or an underlying coronary lesion (12–17).

CACP is provoked by cocaine use through several biological processes, including increased myocardial oxygen demand (by inotropic and chronotropic effects), coronary vasoconstriction; in some cases it is associated with a prothrombotic state with increased platelet aggregation and thrombus formation (5, 6, 18, 19).

The environmental and genetic factors associated with CACP have not yet been described (7). The contribution of cocaine use pattern itself and the effect of other substance use deserve a specific analysis. Thus far, early cocaine use initiation has been associated with a higher risk of lifetime CACP history (11). A specific role of alcohol intake in the occurrence of cardiac events in subjects with cocaine use has been suggested by a pharmacodynamic interaction related to the concurrent use or addition of the two substances, thus acting as cardiovascular risk factors (20). The concomitant use of cocaine and alcohol produces cocaethylene, which has a longer half-life than cocaine and may prolong cardiac effects (21, 22). Tobacco smoking is also a known cardiovascular risk factor and has a significant effect on the general population. Nevertheless, in the analysis of health data regarding cocaine use in the USA, regular cocaine use was shown to increase the risk of myocardial infarction in both subjects with and without concurrent tobacco use disorder, with similar odds ratios (8). On the other hand, no study has considered the concomitant use of opioids as a risk factor for cardiovascular events in cocaine users. However, a previous analysis conducted in the same sample involving patients who used opioids before their first cocaine use, reported significantly less anxiety and tachypsychia while using cocaine than patients who had not used opioids (23), suggesting that opioid use should also be taken into account.

Concerning the hypothesis of genetic variability of CACPs, the literature identifies several genes contributing to the general pathophysiology of vasospasms (5, 6). These genes belong to the NOS signaling pathway, including *NOS3, ROCK2, EDN1, and GUCY1A3*, and have been associated with coronary spasm episodes (24–28). Furthermore, several polymorphisms of *ALDH2*, the gene coding for the enzyme metabolizing acetaldehyde, have also been shown to increase the risk of coronary spasms in East Asian populations (29). *ALDH2* is also the most replicated risk gene for excessive alcohol use (30, 31) and alcohol-related complications (32).

The aim of the present study was to describe the clinical and genetic risk and protective factors of lifetime CACP history in a sample of cocaine users recruited in a substance use disorder care setting, with a specific focus on alcohol, tobacco, and opioid co-occurring use disorder.

## Materials and Methods

### Subjects and Sampling Procedure

The subjects were cocaine users, recruited between 2012 and 2016 in nine urban out- or inpatient specialized treatment centers were they were currently treated for substance use disorders in France (NCT01569347). They were recruited if they used cocaine regardless of whether they met criteria for cocaine use disorder. As European ethnicity was not an inclusion criterion, we *a posteriori* selected subjects with European ancestry by comparing identity by descent to the 1000Genome database, as described in Marees et al. (33).

### Measure of CACP Lifetime History

The records of lifetime CACP events were retrospectively analyzed. First, the subjects were interviewed about their lifetime history of chest pain using the procedure proposed by Edmondstone (34). Those who reported such a history were requested to describe the phenomenology of these events, using the sentence “show me where your pain was and tell me what it felt like.” The chest pain events were rated by the investigator as plausible cardiac origin if they matched with the three Edmondstone's descriptions (“clenched fist to the center of the sternum,” “flat hand to the center of the sternum,” or “both flat hands drawn from the center of the chest outwards”). This method had a sensitivity of 80%, a specificity of 49%, a positive predictive value of 77% and a negative predictive value of 53% (34). Only pain events with both Edmondstone's phenomenology and consistent time relation with cocaine use were retained for the analysis.

The number of lifetime CACP occurrences, pain type (constrictive, oppressive, burn, or stabbing), pain location (retrosternal, laterothoracic, or dorsal), pain intensity, average time of occurrence after the beginning of cocaine use session, average duration of CACP, age at the first CACP occurrence, and history of medical consultation for any of these CACP events were also recorded.

### Sociodemographic Factors and Pattern of Substances Use

Sociodemographic factors were collected at the time of inclusion. These included age, body mass index (BMI), sex, and educational attainment.

The characteristics of lifetime cocaine use and history of other substances were recorded. Lifetime cocaine use was characterized in terms of age at the first use, frequency, preferred routes of cocaine use (intranasal vs. rapid route of use, i.e., smoked and/or intravenous use), ever used cocaine through intravenous administration, and the type of cocaine (crack vs. cocaine hydrochloride) during the worst period of use. Concerning other substances, lifetime histories of use or DSM-IV-TR dependence criteria (35) were recorded for tobacco, alcohol, cannabis, amphetamines, opioids, and lifetime opioid maintenance treatment (OMT) prescriptions.

### Genetic Factors

#### Single Nucleotide Polymorphisms Genotyping

Genomic DNA was extracted from EDTA-treated peripheral blood samples and genotyped using the Illumina Infinium PsychArray24 v1.1 BeadChip, containing 571 054 single nucleotide polymorphisms (SNPs).

#### SNPs Selection and Genetic Quality Control

A focused candidate gene approach was used. *NOS3, ROCK2, EDN1, GUCY1A3*, and *ALDH2* were selected. From these five genes, we retrieved 120 genotyped SNPs from the PsychArray chip. We applied a quality control selection with the following parameters: SNP genotyping rate ≥ 0.05, minor allele frequency ≥ 0.05, and no significant deviation from Hardy Weinberg Equilibrium (*p*-value < 0.001). In the per-individual quality control, we excluded outliers with excessive missing genotyping (>0.05) and heterozygosity (>±3 SD) rates (33). We finally retained 30 out of 120 SNPs (Supplementary Table 1).

### Statistical Analysis

Associations of lifetime CACP history with clinical variables were tested with the chi-square or Student's *t*-test as appropriate, with a specific focus on cocaine use pattern itself and the effect of other substance use. Multivariate analysis of CACP history was performed using logistic regression, with a threshold for entry of *p* < 0.10. Age, sex, lifetime opioid use, and alcohol dependence were added to the model to account for these population-specific factors. Sensitivity analyses according to the first OMT drug ever prescribed as well as the currently prescribed OMT were performed using logistic regression.

In the subgroup of patients with a lifetime history of CACP, the number of CACP occurrences by year of regular cocaine use ratio was calculated. We tested the univariate association between this ratio and each clinical and socio-demographic variable with quasi-Poisson regressions to select variables with a threshold for entry (*p* < 0.10) for a multivariate quasi-Poisson regression. Similarly, age, sex, frequency of cocaine use during the worst period, lifetime opioid use, and alcohol dependence were also added to the model.

For genetic analyses, the association of the SNPs that passed the quality check with (i) lifetime CACP history (chi-square test) and (ii) CACP episodes per year ratio were tested (quasi-Poisson regressions), with a correction for multiple testing using the false discovery rate (FDR) method. We then included genetic factors (*p* < 0.10) in the two previous multivariate analyses. Interaction factors (*p* < 0.10) were retained. Model predictabilities were estimated by McFadden's pseudo-R^2^ and compared with the likelihood-ratio test.

Epidemiological statistical analyses were performed using R software version 3.2. Quality control of genetic data and statistical genetic analyses were performed using PLINK 1.9.

### Ethics

The study was conducted in accordance with the Declaration of Helsinki and French laws on biomedical research. Written informed consent was obtained from all participants. This study was approved by the relevant ethics committee (CPP Ile de France IV) in March 2011.

## Results

### Subjects' Characteristics

The original sample consisted of 418 subjects. Clinical data on lifetime CACP history were available for 316 subjects; among them, 290 were identified as having European ancestry.

Among the 316 subjects, 68 were women (21.5%) and 248 were men (78.5%). The mean age was 37.5 years (SD ± 8.7). The median length of regular cocaine use was 5 years. The frequency of cocaine use during the worst period was daily for 60.1% of the subjects and weekly for 29.4%. Crack use was described by 27.8% of the subjects, and smoked or/and intravenous cocaine use was preferred by 38.3%. Lifetime alcohol and cannabis dependence were observed in 63.2 and 61.2% of the subjects, respectively. Among the 140 subjects (44.4%) with lifetime OMT prescription, 84 were currently on methadone (60.0%), 37 were currently on buprenorphine (26.4%) and 19 had ceased OMT (13.6%). The first OMT prescription was methadone for 47 subjects (34.3%) and buprenorphine for 90 (65.7%). The characteristics of the subjects are shown in Table 1.

**Table 1:** Subjects' characteristics and lifetime substances use pattern (*n* = 316) (*n*, %).

Sex	Male	248 (78.5%)
Age (years)	Mean (SD)	37.47 (±8.65)
	Minimum–Maximum	19–65 years
Body mass index (kg/m^2^)	<21.0	80 (26.1%)
	21.0–24.99	132 (43.1%)
	≥25.0	94 (30.7%)
	Missing	10
Academic attainment	< High school degree	88 (27.8%)
	High school degree	93 (29.4%)
	Graduate degree	135 (42.7%)
Frequency of cocaine use during the worst period	Daily	190 (60.1%)
	1–6 use by week	78 (24.7%)
	Less than weekly	48 (15.2%)
Length of regular cocaine use (year)	Median (Minimum–Maximum)	5 (1–34)
	1st quartile; 3rd quartile	2.5; 11
Crack use (vs. cocaine hydrochloride)	Yes	88 (27.8%)
Preferred route of cocaine use:	Smoked or/and intravenous	121 (38.3%)
	Intranasal (sniffed)	195 (61.7%)
History of intravenous cocaine use	Yes	85 (26.9%)
	Missing	1
Age of cocaine first use (years)	Mean (SD)	23.4 (±7.3)
	Median (Minimum–Maximum)	21.0 years (12–53)
Opioid use and opioid maintenance treatment (OMT)	Never	103 (32.7%)
	Opioid use without OMT	72 (22.9%)
	OMT use	140 (44.4%)
	Missing	1
Amphetamine use during the worst period	Never	124 (39.9%)
	Non-daily use	131 (42.1%)
	Daily use	56 (18.0%)
	Missing	5
Tobacco use	Less than daily use	19 (6.0%)
	Daily use	296 (94.0%)
	Missing	1
Lifetime alcohol use^*^	No dependence	114 (36.8%)
	Dependence	196 (63.2%)
	Missing	6
Lifetime cannabis use^*^	No dependence	121 (38.8%)
	Dependence	191 (61.2%)
	Missing	4
*ALDH2 rs2238151* genotype (*N* = 96, sample restricted to patients with lifetime CACP history)	CC	19 (19.8%)
	CT	34 (35.4%)
	TT	43 (44.8%)
*ALDH2 rs2238151* genotype (*N* = 76, sample restricted to patients with lifetime CACP history and European Ancestry)	CC	9 (11.8%)
	CT	30 (39.5%)
	TT	37 (48.7%)

* *Dependence according to DSM IV -TR criteria*.

### Lifetime CACP History

Of the 316 subjects, 103 (32.6%) reported at least one episode of lifetime CACP history. Among those with a lifetime history of CACP, 86 (87.8%) described several episodes with a median of 10 events per individual. The pain was retrosternal in 57 (60.6%) and oppressive in 49 (50.0%) subjects. Twenty-five (24.8%) consulted a medical doctor after a CACP event. The details are presented in Table 2.

**Table 2:** Characteristics of CACP (*n* = 103).

Number of CACP occurrence	
Median	10
Minimum–Maximum	1–40
1	12 (12.2%)
2–5	19 (19.4%)
6–10	36 (36.7%)
11–20	29 (29.6%)
>20	2 (2.0%)
Missing	5
Pain type	
Constrictive	29 (29.6%)
Oppressive	49 (50.0%)
Burn	5 (5.1%)
Stabbing	15 (15.3%)
Missing	5
Pain location	
Retrosternal	57 (60.6%)
Laterothoracic	30 (31.9%)
Dorsal	7 (7.4%)
Missing	9
Pain intensity between 1 and 10 (*n* = 101)	
Mean (SD)	6.2 (±2.3)
Median	6.0
Minimum–Maximum	1–10
Average lapse of time of occurrence of CACP after cocaine use	
≤10 min	25 (25.8%)
>10 to≤30 min	19 (19.6%)
>30 min to≤1 h	18 (18.6%)
>1 to≤5 h	23 (23.7%)
>5 to≤24 h	10 (10.3%)
>24 to <48 h	2 (2.1%)
Missing	6
Average duration of CACP	
≤10 min	33 (34.4%)
>10 to≤30 min	21 (21.9%)
>30 min to≤2 h	20 (28.8%)
>2 to≤5 h	14 (14.6%)
>5 to≤24 h	6 (6.3%)
>24 to <48 h	2 (2.1%)
Missing	7
Age of first occurrence (years) (*n* = 94)	
Mean (SD)	30.6 (±7.6)
Median	29.5
Minimum–Maximum	16–50
Medical consultation for CACP	
Yes	25 (24.8%)
No	76 (75.2%)
Missing	2

### Clinical Factors Associated With a Lifetime CACP History

In the univariate analysis, subjects with a lifetime history of CACP were significantly younger (36.0 vs. 38.2 years, *p* = 0.034). They also described a higher frequency of cocaine use (*p* = 0.016), amphetamine use (*p* = 0.012), and a preference for rapid route of cocaine use (i.e., smoked and/or intravenous use) during the worst period (*p* = 0.034) (Table 3).

**Table 3:** Analyses of lifetime CACP history (*n* = 316).

	**Cocaine-Associated Chest Pain (CACP) history**					
	**Univariate analysis;** ***n*** **(%) (*****n*** **=** **316)**			**Multivariate analysis (*****n*** **=** **282)**		
	**CACP** ** (*n* = 103)**	**No CACP** ** (*n* = 213)**	***Test p*-value**	**OR**	**OR 95%CI**	***p*-value**
**Sex**						
Female Male	25 (36.8%) 78 (31.5%)	43 (63.2%) 170 (68.5%)	Chi^2^ 0.41	1.06 Ref	0.54–2.04 Ref	0.86
**Age (years);** Mean (SD)	36.0 years (±8.5)	38.2 years (±8.7)	Student 0.034	0.97	0.94–1.01	0.17
**Body mass index (kg/m**^**2**^**)**						
<21.0 21.0–24.99 ≥25.0 Missing	29 (36.3%) 46 (34.8%) 26 (27.7%) 2	51 (63.7%) 86 (65.2%) 68 (72.3%) 8	Chi^2^ 0.41	–	–	–
**Frequency of cocaine use during the worst period**						
Daily 1–6 use by week Less than weekly	72 (37.9%) 23 (29.5%) 8 (16.7%)	118 (62.1%) 55 (70.5%) 40 (83.3%)	Chi^2^ 0.016	3.24 2.02 Ref	1.29–9.33 0.70–6.47 Ref	0.018 0.21
**Crack use**						
Yes No, cocaine hydrochloride only	33 (37.5%) 70 (30.7%)	55 (62.5%) 158 (69.3%)	Chi^2^ 0.25	–	–	–
**Preferred route of cocaine use**						
Smoked or/and intravenous Intranasal (sniffed)	48 (39.7%) 55 (28.2%)	73 (60.3%) 140 (71.8%)	Chi^2^ 0.035	2.33 Ref	1.20–4.65 Ref	0.014
**History of intravenous cocaine use**						
Yes No Missing	35 (41.2%) 68 (29.6%) 0	50 (58.8%) 162 (70.4%) 1	Chi^2^ 0.051	–	–	–
Age of cocaine first use; Mean (SD)	22.7 years (±5.9)	23.7 years (±7.9)	Student 0.24	–	–	–
Length of regular cocaine use (years)	8.29 (±7.9)	8.19 (±7.5)	Student 0.93	1.01	0.97–1.06	0.51
**Lifetime opioid use and opioid maintenance treatment (OMT)**						
OMT use Opioid use without OMT Never Missing	46 (32.9%) 20 (27.8) 37 (35.9%) 0	94 (67.1%) 52 (72.2%) 66 (64.1%) 1	Chi^2^ 0.53	0.35 0.49 Ref	0.15–0.76 0.22–1.05 Ref	9.0 × 10^−3^ 0.072
**Lifetime initiation of cocaine and opioids use**						
Cocaine before opioid Opioid before cocaine or at the same time Missing	57 (30.8%) 44 (34.9%) 2	128 (69.2%) 82 (65.1%) 3	Chi^2^ 0.45	–	–	–
**Amphetamine use during the worst period**						
Daily use Non-daily use Never use Missing	25 (44.6%) 46 (35.1%) 29 (23.4%) 3	31 (55.4%) 85 (64.9%) 95 (76.6%) 2	Chi^2^ 0.012	2.77 2.12 Ref	1.10–7.03 1.06–4.31 Ref	0.030 0.039
Age of amphetamine first use; Mean (SD)	22.3 years (±6.1)	22.1 years (±5.5)	Student 0.82	–	–	–
**Lifetime initiation of cocaine and amphetamine use**						
Cocaine before amphetamine Amphetamine before cocaine or at the same time Missing	61 (28.8%) 39 (39.4%) 3	151 (71.2%) 60 (60.6%) 2	Chi^2^ 0.062	Ref 1.01	Ref 0.52–1.97	0.97
Number of cigarette by day; Mean (SD)	17.4 (13.4)	15.1 (11.3)	Student 0.12	–	–	–
**Alcohol use**						
Dependence No dependence Missing	67 (34.2%) 35 (30.7%) 1	129 (65.8%) 79 (69.3%) 5	Chi^2^ 0.53	1.67 Ref	0.94–3.00 Ref	0.083
**Cannabis use**						
Dependence No dependence Missing	68 (35.6%) 34 (28.1%) 1	123 (64.4%) 87 (71.9%) 3	Chi^2^ 0.17	–	–	–

In the multivariate analysis, age was not found to be an independent risk factor (*p* = 0.17). We observed an association between the lifetime history of CACP and three factors describing the severity of lifetime psychostimulant use: (i) the frequency of cocaine use [daily cocaine use: odds ratio (OR) 3.24; 95% confidence interval (95%CI) (1.29–9.33) vs. less than weekly cocaine use], (ii) the frequency of amphetamine use [daily amphetamine use: OR 2.80; 95%CI (1.25–6.32); and non-daily amphetamine use: OR 2.14, 95%CI (1.15–4.04) vs. no lifetime amphetamine use], and (iii) the preference for rapid route of cocaine use [OR 2.33; 95%CI (1.20–4.64) vs. intranasal cocaine use]. Conversely, receiving or having received a prescribed OMT was inversely associated with lifetime CACP history [OR 0.35; 95%CI (0.15–0.76) vs. no lifetime opioid use]. Overall, the multivariate analysis model, performed for 282 subjects, was significantly different from the null model (McFadden's pseudo-R^2^ = 0.085, *p* = 3.0 × 10^−3^). In sensitivity analyses in the multivariate model, according to the OMT drug prescribed, no significant difference was observed for the first OMT drug prescribed [buprenorphine vs. methadone: OR 0.63; 95% CI (0.25–1.64)], for the current OMT drug prescribed [buprenorphine vs. methadone: OR 0.84; 95% CI (0.30–2.26)], and for OMT continuation vs. OMT cessation [OR 0.50; 95% CI (0.15–1.73)].

### Clinical Factors Associated With a Higher CACP Occurrence per Year Ratio

Univariate analysis was performed on 103 subjects with a lifetime CACP history. The younger subjects displayed a significantly higher ratio (quasi-Poisson regression: β = −0.047; *p* = 4.5 × 10^−3^). None of the factors describing psychostimulant use intensity were associated with a higher ratio. In the multivariate analysis performed for 90 subjects, age remained an independent factor after adjustment for sex, cocaine use frequency, lifetime opioid use, and alcohol dependence [quasi-Poisson regression β = −0.051; 95% CI (−0.085; −0.018), *p* = 4.3 × 10^−3^]. Overall, this second multivariate analysis model was significant (McFadden's pseudo-R^2^ = 0.16, *p* < 10^−3^).

### Genetic Association

Of the 120 selected SNPs, 30 passed the quality control. In the univariate analysis of each individual SNP with lifetime CACP history, the two top SNPs were *rs7658967* and *rs13139571* (*GUCY1A3* gene), with a *p*-value of 0.025 and 0.10, respectively, before FDR correction (after FDR correction: 0.76 and 1.0). The inclusion of these two SNPs in the multivariate model of CACP history did not change the associations observed with cocaine, amphetamine, and opioid use (McFadden's pseudo-R^2^ without SNPs = 0.077 performed in 271 subjects, McFadden's pseudo-R^2^ with two SNPs = 0.089; *p* = 0.54). As the genetic analyses did not identify any significant association between the two *GUCY1A3* SNPs and lifetime CACP history, we did not further adjust for ethnicity.

Similarly, we tested the association of the 30 SNPs that passed the quality check with the CACP occurrence per year ratio. The top SNP was *rs2238151* (*ALDH2* gene) with a *p*-value of 0.017 before FDR correction (and *p* = 0.52, after FDR correction). The risk genotype was CC vs. TT [β = 0.89; 95% CI (0.28–1.50)]. In the quasi-Poisson regression multivariate model of the occurrence ratio performed for 83 subjects, a significant interaction was observed between *rs2238151* and two clinical variables: frequency of cocaine use (*p* = 9.8 × 10^−3^) and alcohol dependence (*p* = 0.026), as well as an interaction with age (*p* = 0.064). The stepwise regression retained the frequency of cocaine use, age, alcohol dependence, *rs2238151* variables, and the interaction terms (McFadden's pseudo-R^2^ = 0.536; *p* < 10^−4^). When restricting the quasi-Poisson regression to the 76 European subjects, the only observed significant interaction (*p* = 4.5 × 10^−3^) was between the frequency of cocaine use and *rs2238151* (McFadden's pseudo-R^2^ = 0.487, *p* < 10^−4^).

## Discussion

A lifetime CACP history was observed in 32.6% of this sample, and 87.8% of patients describing one event had repetitive occurrences. Of note, only a quarter of the affected subjects consulted a medical doctor following a CACP event.

As a summary of the risk factors that we identified, we can state that the frequency of cocaine and amphetamine use and the preference for rapid routes of administration were associated with lifetime CACP history, in contrast to that with a lifetime OMT prescription. Younger age was associated with a higher incidence of CACP. We did not observe a significant effect of tobacco smoking or alcohol dependence. Among the candidate genes that we tested, none reached statistical significance level, but the top SNPs were located on *the GUCY1A3* and *ALDH2* genes.

To the best of our knowledge, this is only the second epidemiological study documenting the prevalence of lifetime CACP history in cocaine users outside of cardiologic or emergency settings, with a larger sample size to enable confirmation of descriptive results and a more powerful analysis of risk factors (11). Thus, our results confirm that both lifetime CACP history and CACP repetitive occurrence episodes are frequent. Regarding recurrences, our results are in concordance with the emergency department data (11–14).

Regarding the lifetime CACP history risk factors that we identified, namely the frequency of cocaine and amphetamine use, and the preference for rapid routes of administration, the analysis strongly suggests that both pharmacodynamic and pharmacokinetic parameters influence CACP. This is in accordance with the results of previous studies. Amphetamine use has also been described as a risk factor for chest pain or acute myocardial infarction (7, 36), independent of cocaine use (37). In addition to the shared acute vasoconstrictor, inotropic, chronotropic, and QT prolongation effects, amphetamine has been specifically suggested to induce chronic coronary lesions and a delayed risk of coronary events not limited to the time of amphetamine use (36, 38, 39). Amphetamine use and frequency of use could also be a marker of severity in cocaine users. However, amphetamine use remains associated with lifetime CACP history in multivariate analysis after adjustment for cocaine use variables. Thus, our data suggest that the intensity of amphetamine and cocaine use are independent lifetime CACP history risk factors that may accumulate in an individual subject.

The negative association of OMT with lifetime CACP history observed in this study is an original result. It was observed in the multivariate analysis after adjustment for frequency and route of cocaine use and amphetamine use, underlining the importance of joint consideration in these multi-users (23). This can be explained by at least two mechanisms. First, opioids have a negative chronotropic effect and decrease the heart rate (40). This action could counteract the chronotropic effects of cocaine and reduce the oxygen requirements of the myocardium. Second, opioids have an analgesic effect and may reduce the perception of heart pain so that users would report less CACP. Although methadone and buprenorphine display different cardiovascular risk profiles (41), no difference was observed between these two drugs for lifetime CACP history in our sample. The difference in lifetime CACP history in opioid users with or without OMT prescription or between OMT drugs prescribed need to be investigated to clarify the effect of this prescription. Especially, the lack of association with opioid use without OMT prescription observed here may be the consequence of a lack of power or be the result of a specific protective effect of steady-state opioid effect of OMT rather that should not be observed with the intermittent use of street opioids.

Age is a known cardiovascular risk factor. A higher CACP history frequency and a higher number of events in younger patients seem paradoxical. First, younger patients may have severity factors that were not captured in the analysis. Second, the year of occurrence of the CACP is not recorded, and it is possible that the increase in cocaine concentration of the products traded on the illicit market or the change in adulterants over time could induce a generation effect (42). In addition, memory bias may differentially affect older patients.

Regarding tobacco smoking, there is probably a ceiling effect in this population, 94% of whom are daily tobacco smokers. In addition, lifetime alcohol dependence as recorded in our sample may have occurred concurrently or non-concurrently with the worst cocaine use period.

For candidate genetic association studies, a lack of power cannot be ruled out. Furthermore, this cohort showed the respective roles of several clinical risk factors that need to be tested independently from one another in specifically designed samples. However, our results show that clinical factors describing the intensity of psychostimulant intake have a larger effect size than individual SNPs in predicting lifetime CACP history.

Our top SNPs were intronic and located on *the GUCY1A3* and *ALDH2* genes. For *the GUCY1A3* gene, among the two identified SNPs in our results, *rs13139571* has been described as being associated with elevated blood pressure (43) and we could not identify any previous literature on *rs7658967*. For *ALDH2 rs2238151*, the CC genotype is protective against a high level of alcohol consumption and alcohol dependence (44); however, it is also a risk factor for head and neck cancers among heavy drinkers (45). This is consistent with the role of acetaldehyde metabolism in alcohol toxicity.

This study had several limitations. First, the assessment of lifetime CACP history and other factors was carried out by a cross-sectional and retrospective interview, prone to bias. No continuous measurement of substance use has been conducted. Second, the CACP occurrences were self-declared. Thus, they were not investigated by electrocardiogram, coronarogaphy, troponin levels, or magnetic resonance imaging, to prove the cardiac origin of the chest pain (aortic dissection, crack lung, rhabdomyolysis). However, we used a rigorously standardized interview based on the anamnesis elements presented by Edmondstone (34) and excluded events not chronologically related to cocaine use. Among the 25 patients who reported having a medical consultation for CACP, the corresponding medical record was not available; especially the type of complementary examinations performed in the diagnosis process was unknown. Third, cognitive impairments were not an exclusion criterion. Mild to moderate cognitive impairments are frequent in cocaine and amphetamine users and notably in relation with the intensity of use (46), possibly leading to memory bias. Fourth, psychiatric comorbidities were not an exclusion criterion. They are associated with the prevalence of cardiovascular disease, a higher rate of cardiovascular risk factors and other risk factors shared with cocaine use, such as unhealthy lifestyle and social deprivation (47), possibly leading to a confounding bias. Fifth, our study population was composed of cocaine users currently in care. They displayed a high frequency of cocaine use, and most of them met the criteria for cocaine dependence, in addition to being multiple substance users. Our study did not cover “hidden populations,” such as subjects with occasional use. Fourth Sixth, the presence of lifetime CACP history may be one of the factors motivating entry into treatment. Fifth Seventh, our retrospective interview assessed lifetime CACP events. We can only hypothesize that most of them occurred during the worst period of cocaine and amphetamine use. We only showed lifetime associations, and the causality of the identified risk factors describing cocaine and amphetamine use intensity (frequency, rapid route of administration) needs to be investigated in prospective studies. Eighth, the sample size implies a lack of power for genetic association analysis.

The strengths of our study are the use of a rigorous standardized interview to characterize CACP, and a sample size that is sufficient to provide evidence of several risk factors with acceptable power in cocaine users. Including a genetic association study is also a strength, as the results highlight the major role of the clinical factors relative to genetic factors in predicting lifetime CACP history. Lastly, our study could serve to calculate the number of subjects included in specific studies designed to test the supplementary risk conferred by *the ALDH2* risk genotype.

There are no validated treatments for primary prevention or recurrence. The use of beta-blockers is controversial. Long-term management includes the reduction of associated cardiovascular risk factors and abstinence of cocaine use (48). Our results suggest that harm reduction approaches may be of interest to reduce CACP occurrence, especially in patients with a previous history of CACP. This could include motivational or behavioral therapies aimed at reducing alcohol and cocaine use or changing the route of cocaine use in patients with a previous history of CACP. Optimization of OMT in patients with opioid use disorder, cocaine use disorder, and CACP history should be investigated. This could include dose optimization and diverse OMT formulation proposals, including long-term depot treatments. Future research should aim to reduce cardiac events and mortality in patients with cocaine use disorders.

## Conclusions

This study confirmed that recurrent CACP events are prevalent among cocaine users. It identified lifetime CACP history risk and protective factors composed of variables describing the intensity of stimulant use and highlighted an original negative association with OMT. These factors open an avenue for targeted prevention and specific harm reduction policies targeting polydrug use and administration patterns to reduce damage associated with cardiac events. The role of OMT deserves further study.

## Data Availability Statement

The datasets presented in this article are not readily available because they are the property of the Direction de la Recherche Clinique et de l'Innovation (DRCI) of Assistance Publique - Hôpitaux de Paris. Requests to access the datasets should be directed to the DRCI.

## Ethics Statement

The studies involving human participants were reviewed and approved by CPP Ile de France IV. The patients/participants provided their written informed consent to participate in this study.

## Author Contributions

VC-D and FV drafted of manuscript. VC-D, LN-S, FV, and VB analyzed the data. All PSYCHOCOKE investigators have contributed to the data collection. J-LL, GB, FV, and VB wrote the protocol. All authors revised and approved the manuscript.

## The PSYCHOCOKE Investigators

Florence Vorspan, Maeva Fortias, Jean-Pierre Lépine, Georges Brousse, Ingrid de Chazeron, El-Hadi Zerdazi, Jean-Baptiste Trabut, Beatriz Belforte, Philippe Coeuru, Delphine Moisan, Arnaud Plat, Philippe Batel, Alice Deschenau, Olivier Cottencin, Aurelia Gay, Philippe Lack and Anne-Laure Pelissier-Alicot.

## Conflict of Interest

In the past 5 years, FV had congress fees paid by CAMURUS AB (2018, 2019), and RECORDATI (2020). FV gave a single lecture for RECORDATI (2020) and served in advisory boards for CAMURUS AB (2019), and ACCORD HEALTH CARE (2021). All payments were made to a research entity and not directly to FV. The remaining authors declare that the research was conducted in the absence of any commercial or financial relationships that could be construed as a potential conflict of interest.
