# Supplementary material for: Risk and Protective Factors of Lifetime Cocaine-Associated Chest Pain
Source: Front Psychiatry. 2021 Jul 21;12:704276. doi: 10.3389/fpsyt.2021.704276 (PMC8335401; doi:10.3389/fpsyt.2021.704276)
Supplement: Supplementary file 1 [file Table_1.DOCX]

**Supplemental online material**

Supplementary table 1:

| Gene | Single Nucleotide Polymorphisms |
| --- | --- |
| *GUCY1A3* | *rs13139571, rs7685675, rs7692387, rs9993460, rs3796585, rs10019835, rs61736997, rs2170646, rs2101180, rs149412037, rs7658967, rs2625276, rs3796579, rs116089180* |
| *ROCK2* | *rs978906, rs978906, rs7598032, rs2230774, rs6716817, rs6759490, rs10929728, rs10203916* |
| *NOS3* | *rs3918184, rs891511, rs1808593, rs7830* |
| *EDN1* | *rs1629862, rs5370* |
| *ALDH2* | *rs2238151, rs11066028* |
